# Show Me the Meaning of Being Lonely: Pathways from Cognitive Functioning to Loneliness and Social Isolation

**DOI:** 10.1093/geroni/igaf122.3121

**Published:** 2025-12-31

**Authors:** Lydia Grenko, Elizabeth Anquillare, Caroline Collins-Pisano, Andrew Lac

**Affiliations:** University of Colorado Colorado Springs, Colorado Springs, Colorado, United States; University of Colorado Colorado Springs, Colorado Springs, Colorado, United States; University of Colorado Colorado Springs, Colorado Springs, Colorado, United States; University of Colorado Colorado Springs, Colorado Springs, Colorado, United States

## Abstract

Social isolation and loneliness (SI/L) are increasingly prevalent in older adults and connected with worse outcomes in later life. Previous research has focused on the effects of SI/L on cognitive functioning in older adults. However, fewer studies have investigated the pathways from cognitive impairment to SI/L. Mental health and social networks may serve as intermediate constructs that help to account for the link between cognition and SI/L. The present study examined the predictive pathways from cognitive functioning to SI/L through mental health and quality of relationships using data from the National Social Life, Health and Aging Project (NSHAP). Community-dwelling older adults (N = 4,337, Mage=67.63, SD = 10.95) completed a cognitive assessment and mental health and social functioning surveys. Structural equation modeling produced satisfactory fit indices, CFI=.97, TLI=.96, RMSEA=.03. Specifically, higher cognitive functioning predicted higher perceived quality of social relationships (β=.22, p<.001), fewer mental health symptoms (β=-.25, p<.001), greater frequency of social interaction (β=.22, p<.001), and higher self-reported loneliness (β=.18, p<.001). Additionally, higher quality of social relationships predicted less social isolation (β=.22, p<.001) and loneliness (β=-.14, p<.001), whereas greater mental symptoms predicted greater social isolation (β=-.13, p<.001) and loneliness (β=.66, p<.001). Less loneliness in the context of lower cognitive functioning may reflect a sense of subjective closeness with fellow older adults experiencing normative cognitive change. Older adults experiencing cognitive decline may benefit from interventions addressing mental health symptoms, fortifying social supports, and compensatory cognitive strategies to improve social functioning and overall well-being

